# Functional Synthetic Biology

**DOI:** 10.1093/synbio/ysad006

**Published:** 2023-04-08

**Authors:** Ibrahim Aldulijan, Jacob Beal, Sonja Billerbeck, Jeff Bouffard, Gaël Chambonnier, Nikolaos Ntelkis, Isaac Guerreiro, Martin Holub, Paul Ross, Vinoo Selvarajah, Noah Sprent, Gonzalo Vidal, Alejandro Vignoni

**Affiliations:** Systems Engineering, Stevens Institute of Technology, 1 Castle Point Terrace, Hoboken, 07030, NJ, USA; Intelligent Software & Systems, Raytheon BBN Technologies, 10 Moulton Street, Cambridge, 02138, MA, USA; Molecular Microbiology, Groningen Biomolecular Sciences and Biotechnology Institute, University of Groningen, Nijenborgh 7, 9747 AG, Groningen, The Netherlands; Centre for Applied Synthetic Biology, and Department of Biology, Concordia University, 7141 Sherbrooke Street West, Montréal, H4B 1R6, Québec, Canada; Department of Biological Engineering, Massachusetts Institute of Technology, Cambridge, 02139, MA, USA; Specialized Metabolism research group, Center for Plant Systems Biology, VIB-Ghent University, Technologiepark 71, Zwijnaarde, 9052, Belgium; iGEM Foundation, 45 Prospect Street, Cambridge, 02139, MA, USA; Delft University of Technology, Van der Maasweg 9, 2629 HZ, The Netherlands; BioStrat Marketing, 9965 Harbour Lake Circle, Boynton Beach, FL, 33437, USA; iGEM Foundation, 45 Prospect Street, Cambridge, 02139, MA, USA; Department of Chemical Engineering, Imperial College London, South Kensington Campus, Exhibition Road, SW7 2AZ, UK; Interdisciplinary Computing and Complex BioSystems (ICOS) research group, School of Computing, Newcastle University, Devonshire Building, Devonshire Terrace, NE1 7RU, Newcastle Upon Tyne, UK; Synthetic Biology and Biosystems Control Lab, Instituto de Automatica e Informatica Industrial, Universitat Politecnica de Valencia, Camino de Vera s/n, 46022, Valencia, Spain

**Keywords:** Synthetic Biology, Engineering, Design, Reproducibility, Collaboration

## Abstract

Synthetic biologists have made great progress over the past decade in developing methods for modular assembly of genetic sequences and in engineering biological systems with a wide variety of functions in various contexts and organisms. However, current paradigms in the field entangle sequence and functionality in a manner that makes abstraction difficult, reduces engineering flexibility and impairs predictability and design reuse. Functional Synthetic Biology aims to overcome these impediments by focusing the design of biological systems on function, rather than on sequence. This reorientation will decouple the engineering of biological devices from the specifics of how those devices are put to use, requiring both conceptual and organizational change, as well as supporting software tooling. Realizing this vision of Functional Synthetic Biology will allow more flexibility in how devices are used, more opportunity for reuse of devices and data, improvements in predictability and reductions in technical risk and cost.

## 1. Introduction

Over the past decade, synthetic biologists have made great strides in the engineering of biological systems. One vision that has served as something of a roadmap is the model articulated by Endy (11) that comprises four levels of increasing abstraction: DNA, parts, devices and systems. This model envisions the development of standards for defining the interfaces of parts and devices and for capturing and sharing information about these interfaces. Standard interfaces would in turn enable a decoupling of design at higher levels of abstraction from the implementation details of lower levels, allowing biological systems to be designed primarily in terms of the function of devices and thereby obviating the need for designers to have a detailed understanding of the specific parts and DNA used to implement those systems.

Consistent with this model, the field has developed a plethora of basic parts such as promoters, terminators, coding sequences and functional RNAs, which can be combined into composite DNA sequences through a variety of standardized assembly methods (e.g. (12,14,18,30,32)) or low-cost nucleic acid synthesis (6). The field has also made great strides toward enabling functional abstractions by producing families of biological devices that effectively produce a variety of well-defined sensing, communication or computational functions (e.g. (5,8,13,16,17,23,28,33)). Enabling technologies for decoupling have been developed in the form of a number of methods for insulating devices from context (e.g. (7,20,22)). The ability to operate abstractly at a functional level has been demonstrated through a number of methods for characterizing and predicting the behavior of devices (e.g. (1–3,8–10,17,23,25,27,28,31)). Finally, a standard has been developed, the Synthetic Biology Open Language (SBOL), that can capture both the sequence and function information needed to represent parts, devices and systems (26; 21).

Despite this progress, however, significant challenges remain in the practice of synthetic biology at a systems level. Specifications for parts and devices are typically unavailable, incomplete or inconsistent. Likewise, little information is generally provided regarding interfaces, functionality or host context, and such information is rarely available in a tool-friendly format. This leads to significant difficulty in searching for appropriate parts or devices to use, in adapting parts and devices from their original context for use within a new project and in predicting the behavior of even the most basic systems from information available about the components used to create them. These difficulties in turn lead to significant time requirements and technical risk to achieve even modest engineering goals.

Under these conditions, engineering success can certainly be achieved, as illustrated by the many billions of dollars of industrial impact from synthetic biology, but it is slow and costly to do so. On the other hand, if engineering could be made simple and predictable, even just for systems comprising small numbers of devices, it would radically lower cost, reduce barriers to access, increase democratization and unleash a wave of innovative applications of synthetic biology by small organizations tackling local problems.

We contend that the primary barrier to achieving this vision is no longer biological, given the many advances that have been achieved. Rather, we argue that the problem is one of knowledge synthesis and organization: given the complexity of biological systems, no practitioner can be expected to even be aware of all of the relevant parts, methods and models, let alone have the detailed expertise in all of them to use and combine them effectively in practice. How can then the advances and expertise dispersed across the synthetic biology community be marshaled in order to enable practitioners to effectively utilize them in their engineering projects?

**Figure 1.**
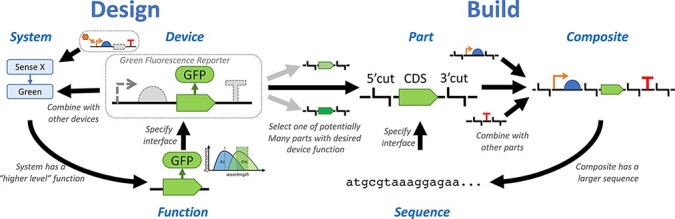
Abstraction layers in a function-centric view: system design focuses on biological functions, which are abstracted to produce ‘devices’ by adding a description of an interface, predicted range of behavior and operational context. Devices may then be combined to produce a multi-device system. This system may be described as having a function of its own at a ‘higher level,’ and the system may be abstracted as a new ‘higher-level’ device with a specified interface that does not depend on knowing all details of its implementation. Actually, building the system requires selecting sequences for each device and composing those sequences (e.g. via direct synthesis or assembly reactions) to form a complete implementation of the system. Each device may have multiple different options for how it can be implemented with ‘good enough’ parts, where parts are sequences with a specified interface for combining them to build composite sequences, which also may in turn be abstracted into higher-level parts.

We propose that in order to meet this challenge, synthetic biology should engage in focused efforts to develop standards-enabled engineering at the functional level (i.e. the composition of devices into systems). Specifically, a Functional Synthetic Biology approach focuses on

- descriptions of behavior over descriptions of structure,
- predictability and flexibility over optimization of function and
- risk reduction over novelty.

A focus on behavior means a that biological component’s structure (i.e. genetic sequence) should be able to be changed and improved without damaging the functionality of a system that includes it. A focus on predictability means identifying classes of changes that are unlikely to damage functionality, and a focus on flexibility means valuing the breadth of such classes of changes when developing biological components. A focus on risk reduction means recognizing that there are many failure modes that can impair the functionality of a biological system and that there is value in capturing knowledge about failure modes (and how to avoid them) in the form of automation tools and machine-readable component specifications.

Together, these approaches will enable the community of synthetic biologists to more effectively share successes and avoid failures. In the following, we will expand on each of these three goals –describing behavior, predictability and flexibility and risk reduction –and lay out a roadmap for achieving these goals over the course of the coming decade.

## 2. Function-centric design descriptions

Current representations and software tools in synthetic biology are mainly sequence-centric, meaning that the first-class objects of a design are sequences—typically DNA, though sometimes RNA or amino acids. Information about function, if it appears at all, is provided as supplemental annotations or in metadata descriptions. Many design representations (e.g. FASTA , GenBank, GFF) reinforce structure-centric description by providing no way of expressing functional information without reference to a fully specified sequence.

In practice, however, synthetic biologists tend to think about designs more in terms of function. Consider, for example, green fluorescent protein (GFP) as a reporter of transcriptional activity (left half of Figure 1). A user of GFP does not typically think about the actual sequences and would be unlikely even to recognize either the nucleic acid or amino acid sequences for GFP if they saw them. Instead, they are likely to think about a functional relationship between a coding sequence that produces a GFP protein, which in turn will fluoresce with a predictable excitation and emission behavior. This is a fully coherent notion of biological function, independent of sequence, and is the typical subject of discussions and diagrams regarding design.

Also associated with the notion of function is a concept of an interface to that function (e.g. embedding the GFP in a transcriptional unit whose activity is to be reported) and of the type of environments where the function is expected to behave as predicted (e.g. aerobic environments with relatively neutral pH across a broad range of cell types). For Functional Synthetic Biology, then, we will define a device as a function that has been associated with a precise description of an interface and a context for its operation. This information is sufficient to combine a device together with others to implement a biological system (e.g. to sense some condition and report it via green fluorescence). Finally, the function of that system may be itself described and possibly abstracted into a higher-level device by identifying its interface and context for operation.

To actually implement a device or system, of course, a specific sequence must be identified. Typically, there are many sequences that may suffice to implement any given device. GFP, for example, at the time of this writing has 160 different amino acid sequences on FPbase (19). Reverse translation of any of these amino acid sequences further produces many distinct nucleic acid sequences, all of which encode the same amino acid sequence. These alternatives are not identical, of course, and likely, some will not be functional at all. Still, a great many can be expected to be sufficiently good implementations of a Green Fluorescence Reporter device.

As with devices, we often need to combine sequences with other sequences in order to form the composites that implement systems, either through assembly protocols or by directly synthesizing the composite sequence. The right half of Figure 1 shows this parallel abstraction hierarchy, in which a part is defined as a sequence that has been associated with a ‘build interface’ that precisely describes how it can be combined with other parts to form composite parts (e.g. restriction enzyme cut sites for use in assembly reactions, homology sites for a recombination-based insertion). Parts may then be combined to form composites, and those composites may in turn be abstracted into higher-level parts by identifying interfaces for further combination.

**Figure 2.**
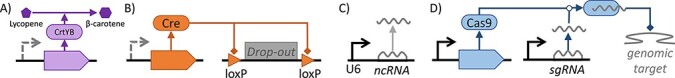
Other examples of functional devices: (A) the CrtYB enzyme catalyzes the production of beta-carotene from lycopene, (B) excision of a drop-out sequence using Cre recombinase targeting loxP sites, (C) constitutive expression of non-coding RNA using a U6 promoter and (D) CRISPR-based gene editing with constitutive expression of Cas9 and sgRNA. The ‘interface’ by which each device connects to other devices is shown in gray.

This approach to defining functional devices can be applied to any well-described biological function: Figure 2 shows other examples of functional devices, some of which would necessarily need to be implemented using multiple parts. Note, however, that the level of specificity that will prove useful to capture functional representations remains an open question. For example, a ‘Green Fluorescence Reporter’ might be defined with stricter or looser constraints on excitation and emission properties or even as far as a generic ‘Fluorescence Reporter’ covering the entire spectrum. There may not even be a single ‘right’ answer to the question, as some use cases will benefit from more predictability, while others benefit more from a higher degree of flexibility. Pragmatically, however, we may reasonably expect that it will be useful to scope functional representations to align well with how practitioners think about and discuss devices.

Indeed, this separated hierarchy is *de facto* what is already in informal use throughout much of the community. From whiteboards to journal articles, synthetic biologists typically communicate primarily in terms of function. Sequences, meanwhile, are often selected arbitrarily or inherited from prior projects via a shared laboratory freezer and, in publications, are often relegated to supplementary information. Even in many cases where synthetic biologists are operating at a low level on sequences, such as selecting sites for an assembly plan, functional concepts play a major role in thinking and communication (e.g. ‘EcoRI cut site’ vs. ‘GAATTC’), as indicated by the typical priority given to displaying functional annotations in design tools, plasmid viewers, talks and figures.

This informal functional view of synthetic biology has been so pervasive that early synthetic biology publications often did not even include the sequences of the constructs used (24). While the field has improved from this state, sequence information is still often relegated to supplemental information, often in formats such as raster graphics or PDFs where the information is difficult to extract. Even when sequence information is included, it is often incomplete and lacking in context or annotation that would help map sequence information to function. Meanwhile, representations and tooling still deal almost exclusively with sequences, with matters of function often handled only in the heads of the practitioners or through non-standardized annotations and bespoke tooling.

Functional Synthetic Biology proposes that we explicitly recognize the distinction between sequence and function and then explore its consequences in order to improve our representations, tooling, engineering approaches and collaboration strategies. Furthermore, since synthetic biologists are already often communicating in this manner, we argue that observing the ways in which synthetic biologists already tend to abstract and cluster functional notions in their communications (e.g. clustering fluorescent reporters into ‘colors’ like ‘green,’ ‘blue’ or ‘red’) provides a good starting point for creating useful functional devices.

## 3. Predictability and flexibility

The importance of predictability and flexibility as design requirements is heightened when a device’s functional description is divorced from its instantiation as a composite of discrete parts. Implementation of a function, whether at the level of a single device or a composite system, depends on identification of an appropriate part or sequence. Moreover, there are typically many parts or sequences that could potentially provide the behavioral properties that are sought.

The typical sequence-centric approach to engineering has tended to approach characterization of parts through questions of the form ‘how does this part behave?’ The questions that a function-centric approach tries to answer, on the other hand, are of the form: ‘is this part’s behavior good enough to be an implementation for that device?’ This latter form of question cannot be answered without considering what ‘good enough’ means in terms of the function of a device.

For example, a Green Fluorescence Reporter device turns transcriptional activity into a strong fluorescence signal. How strong is strong enough? The answer is determined by the signal strength required to discriminate various levels of expression from background with a given class of instrument (e.g. plate reader or flow cytometer), which in turn determines the flexibility of the specifications for the device instantiation, i.e. what types and degrees of imperfections can be tolerated. Device context must also be recorded in the specification, as any GFP coding sequence will fail to produce strong fluorescence if it is placed in the wrong operational context (e.g. under anaerobic conditions, with an incompatible 5’ UTR, or in an incompatible host). For example, if the device is represented in SBOL (21, 26), aerobic and temperature conditions can be recorded using measures on the system and host compatibility as a containment constraint, indicating that the system components are contained in cells with belonging to a taxon for the compatible host range. Useful device specifications thus require at least some predictions about device behavior.

There are an inherent tension and interplay between predictability and flexibility. Any device’s instantiation can be rendered impossible by specifying a degree of precision that is excessively high or an operational range that is too broad, e.g. looking for a Green Fluorescence Reporter that always yields exactly the same number of molecules in wildly different cell types. Likewise, a device can be rendered inoperable if the constraints placed on values are too lax (e.g. accepting a red fluorescent protein as a legitimate implementation of a Green Fluorescence Reporter, which then fails to produce fluorescence in the desired emission range) or if the operational range applied is too tightly constrained (e.g. predicting that the device will work only in the exact construct where it was characterized). Success in designing and building engineered systems depends on finding a middle ground where practitioners can easily locate the devices and parts they need to realize a system and also to predict the outcome that system will generate with a satisfactory degree of reliability and accuracy.

There is likewise a tension between these goals and the desire to obtain the best performance from a device. For example, in selecting a part to realize a Green Fluorescence Reporter device, it may be desirable to select a less bright GFP variant that is better understood (thus more predictable), known to operate in a wider range of organisms or available in a preferred assembly format. Ultimately, this is a matter of trust and focus: the more that a practitioner can trust the predictability of the less interesting parts of their system (e.g. the Green Fluorescence Reporter), the more they can focus on their primary goals (e.g. improvement of a novel metabolite sensor whose activity is being reported).

Navigating the relationship between flexibility, predictability and optimization of function is in general a challenging and unresolved problem in all engineering fields and particularly so for biology. Nevertheless, there are a number of bioengineering tools, such as GFP, that are in common use precisely because they are reasonably effective at producing reasonably predictable behavior across a fairly flexible range of applications and operating conditions. Complementarily, there are operating conditions that are known to be unworkable, such as using GFP in an anaerobic environment. Practitioners who have applied these tools have acquired a great deal of pragmatic knowledge about their range of flexibility and the conditions under which their behavior can be predicted. Some of this accumulated knowledge has found its way into scientific publications, but much of it is still communicated only through informal channels and by word of mouth.

Functional Synthetic Biology proposes that we should begin capturing such knowledge in device specifications. Prior work on predictive modeling (e.g. (9,23,27)) and reproducibility (e.g. (1,2,25)) can provide initial information for some common devices. Similar information can be captured for other devices by collecting it from experts and the literature or by conducting similar studies. The critical element for effective sharing of such information is for measurements to be made in compatible reproducible measurements grounded in SI units, such as cell count (rather than O.D.), molecules of equivalent fluorophore per cell (rather than arbitrary or normalized fluorescence) (1) or kilocalories/mol (rather than relative affinity) (27).

Knowledge captured in SI-based units allows information about independently developed devices to be brought together, e.g. to determine which of a set of constitutive promoters will drive expression of an enzyme at a desired level or to determine the concentration of a sensed analyte from a fluorescent reporter by connecting the model of the sensor to the model of the reporter it is driving. Such knowledge may also be applied, if desired, in optimization processes, particularly multi-objective optimization that can assign value to flexibility (e.g. (4)). Flexibility and predictability of devices may also be improved by applying methods for insulating devices from context (e.g. (7,20,22)), which will have the effect of simplifying the required models.

In sum, the focus on the functionality of a device rather than the sequence of a part reinforces the need for better documentation that can usefully describe the behavior, context and constraints of a genetic object in the same way that other engineering disciplines seek to describe the specifications of a component. It also drives a need for characterization focused on improving the understanding of flexibility and predictability, especially with respect to the composition of devices into systems. Explicitly studying and recording such information in a tool-friendly manner will allow the information to be shared and redistributed more broadly and will allow practitioners to more readily benefit from knowledge and advances produced by others in the field.

## 4. Collaborative reduction of technical risk

Decoupling functional specifications from part sequences also allows new approaches to collaboration and information sharing that can reduce the risk of failure in engineering synthetic biology systems. Here, the key idea is that changes in parts do not necessarily entail changes in devices or vice versa.

When a part is improved, the new version results may still meet the same device specifications that the old version did and thus be able to be substituted as an implementation for that device. For example, a GFP part might be codon optimized to produce the protein more efficiently, or have a restriction site eliminated to make it compatible with a wider range of assembly methods. If the new version of the part still satisfies the Green Fluorescence Reporter device specification, then it can be reasonably predicted that systems using the old version can be upgraded to use the new version. Likewise, a device may be improved with models that better predict its interactions with other devices or enhanced with better documentation of its expected operational range, without affecting the sequence of parts that implement the device.

This decoupling offers a new means of capturing expert knowledge, in the form of curated collections of devices and the recommended parts to implement them. For example, a typical synthetic biologist should never need to concern themselves with the 160 different variations of GFP in FPbase, let alone other green proteins like ZsGreen or mNeonGreen. Instead, they should be able to determine their intended operating range (e.g. *Escherichia coli* DH5*α* in M9 media at 30–37 ^°^C), select a Green Fluorescence Reporter device that operates in that range and then implement it with any of the parts that are currently recommended by experts as a good implementation for that device.

If better parts become available or a problem is detected with one of the current parts, then all that needs to be changed is the recommendation. As long as the functional characteristics of the newly recommended part can be assessed to remain within the Green Fluorescence Reporter device’s range of predictability, any system using the device should be able to be safely updated to use the new part. Indeed, such an upgrade recommendation has already made an appearance in the scientific literature for red fluorescence, when the developers of mCherry suggested phasing out its predecessor, mRFP1, given mCherry’s improved ‘higher extinction coefficient …, tolerance of N-terminal fusions and photostability’. (29)

Assessing the potential impact of a change to a part or device, however, is often not clear-cut or straightforward. If a model is not sufficiently accurate, there is a risk that changes assessed as safe will instead produce unwanted side effects or even result in a system-wide degradation. The risk that might result from adopting a recommendation must be balanced against the risk and costs associated with ignoring a recommendation from experts who are likely to be more familiar with the specialized matter at hand and better able to sort through the many possible implementations of the device. Ultimately, then, this is a question of building trust around changes to complex systems with implications that are difficult to predict.

The software engineering community has faced a very similar problem of managing technical risk and building trust around changes with difficult-to-predict systems implications. That community has addressed its analogous challenge with a now-mature ecosystem of tools and collaborative processes (often collected under the name ‘agile software development’) for managing the development of complex systems.

Foundational to these processes are distributed version control systems (e.g. Git) that afford communities of experts a convenient way to organize, share and maintain packages of information. These are already used in the bioinformatic community to manage knowledge collections such as the Systems Biology Ontology, Sequence Ontology and Gene Ontology.

**Figure 3.**
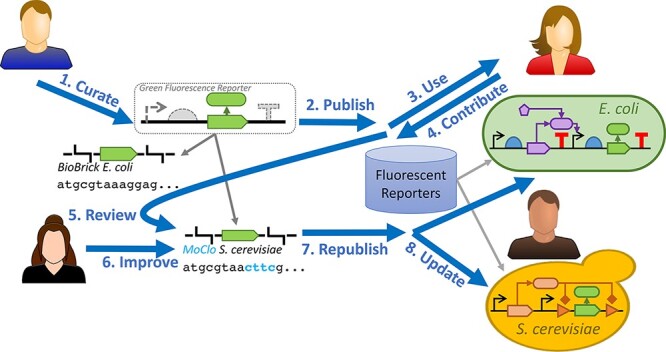
Decoupling function and sequence enables the development of a collaborative ecosystem for distributing, using and improving biological devices. In this vision, (1) an expert curates a device and a set of parts that can implement the device, e.g. a green fluorescence reporter and a BioBricks-compatible coding sequence optimized for *E. coli*. The expert then (2) publishes the device and parts into a collection where they can be discovered by other synthetic biologists, e.g. version 1.0 of a collection of recommended fluorescent reporters. Synthetic biologists (3) obtain the device from the collection and put it to use in various contexts, e.g. as using the green fluorescence reporter as part of a small-molecule sensing system. Those practitioners may (4) contribute back ‘patches’ to improving the device, e.g. improved characterization data or adaption to another context such as adding an implementation with a MoClo-compatible part optimized for *S. cerevisiae*. These contributions are then (5) reviewed by either the original expert or others helping to maintain the collection, e.g. checking that the claimed MoClo-compatible part does in fact pass a compatibility check. The experts may similarly (6) contribute their own improvements to the collection as well. All of these improvements are then (7) made available when the collection is republished as a new version, e.g. version 1.1 of the collection of recommended fluorescent reporters. Finally, (8) the device users receive the benefits of these improvements by updating to the newest version, e.g. better predictions of fluorescence in *E. coli* and use of green fluorescent reporting in *S. cerevisiae* systems.

**Figure 4.**
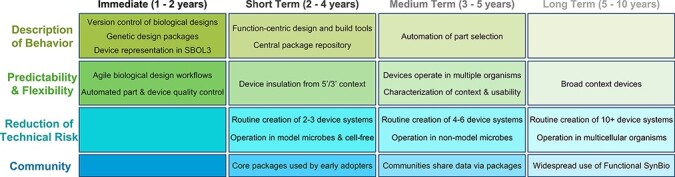
Suggested roadmap of goals, from immediate to long-term, for achieving Functional Synthetic Biology. The first row describes software capabilities for describing behavior and using those descriptions to increase automation in design-related workflows. Next are goals related to increasing the predictability and flexibility of devices. The third row describes how reduction of technical risk (leveraging behavior descriptions and increased predictability and flexibility) will enable routine creation of incrementally larger systems across a growing range of organisms. The final row describes anticipated growth of a functional synthetic biology community building on and amplifying these goals.

Functional Synthetic Biology can take advantage of these same mechanisms to curate collections of devices, collaboratively maintain them and reduce technical risk related to updating these collections. Figure 3 illustrates the type of interactions that can be enabled. In this vision, an expert curates a device such as a Green Fluorescence Reporter, along with options for implementing the device, such as several parts containing a coding sequence for GFP. This device is aggregated with others into a fluorescent reporters collection, which can be published as a package in a catalog where it can be shared with other synthetic biologists.

In the course of applying the device in various contexts, synthetic biologists will undoubtedly identify ways to improve the collection. These improvements might include expanding or sharpening characterization, augmenting context tolerance information, clarifying documentation or enhancing the design of the parts that implement the device.

Distributed version control can make it easy for users to contribute their improvements back as suggested changes to the collection. Agile software tooling can support the curation of such contributions, making it simple for the maintainers of a collection to review and discuss a contribution, request changes where needed and apply automation-assisted validation checks for quality control. Once incorporated, improvements can be taken up in a new version of the collection that is made available to the community when the collection is republished. Users can then take advantage of the benefits these curated improvements provide simply by updating their copy of the collection to the latest version.

When implemented well, the transparency and checking in such processes can help build trust within a community of users and maintainers. The mechanisms of distributed version control also help to sustain an open marketplace for competition between packages of information and their attendant processes and maintainers. Just as in the software world, Functional Synthetic Biology packages will compete on the basis of not just technical efficacy but also their trustworthiness, reliability, ease of use and responsiveness to user needs.

## 5. A roadmap to functional synthetic biology

In this section, we propose a multi-year roadmap for realizing the vision for Functional Synthetic Biology set out earlier. This roadmap foregrounds the vision’s most consequential aspects and specifies the timeframes within which we predict that these aspects can reasonably be achieved (Figure 4).

Some aspects of the vision that the roadmap accounts for are well-defined and can be achieved with technologies and techniques that are available today. They simply need to be adapted and applied. For example, an integrated representation of the part and device hierarchies from Figure 1 can already be implemented using the SBOL 2 (26) or SBOL 3 (21) standards. Alternatively, devices could also be represented with a modeling language such as SBML (15) and then associated with parts represented in GenBank or GFF. In either case, a modern version control system such as Git can be employed to manage and disseminate the genetic design files generated. Another advantage of such a version control system is that it supports mature agile software workflows (e.g. trunk-based development) and tooling (e.g. continuous integration) that foster collaboration and enable quality control automation.

Short-term targets for development include basic parts such as constitutive promoters, terminators, sensors and reporters, as well as regulatory insulation (e.g. (7,20,22)) and families of transcriptional computing devices (e.g. (5,8,13,16,23)). Developing a collection of design packages covering these elements, along with function-centric design and build tools, should enable routine engineering of 2-3 device sense/compute/actuate circuits in model microbes and cell-free systems. The core packages that result from these initial engineering initiatives can be made available through a central repository serving as a rendezvous point for early adopters to discover and make use of these packages.

As this Functional Synthetic Biology ecosystem matures and expands, practitioners will undoubtedly enhance the performance, flexibility and reliability of the parts, add new packages focused on their own areas of interest and expand the functionality of existing packages by making incremental upgrades to them. The complexity of systems that can be routinely engineered will increase, as will levels of automation.

The ability to support more effective sharing of data will then facilitate better understanding and characterization of operational context, expansion into non-model organisms and the creation of more context-agnostic devices that can operate effectively in multiple types of chassis. Over the longer term, we anticipate a widespread adoption of Functional Synthetic Biology, driven by the standardization of complex biological systems engineering, the development of extremely flexible devices and extension even to effective operation *in vivo* in complex multicellular organisms.

Following this, roadmap will also entail tackling non-technical challenges associated with incentives. For example, an initial investment in time and resources is needed from experienced practitioners before the community can benefit, and the people investing will not necessarily be the ones who most benefit. In light of the difficulties that were involved in even obtaining DNA sequence information from scientific publications (24), we can expect that current academic incentives will not be sufficient on their own to motivate widespread investment in the curation and publication of functional information. There will also be questions around governance and degree of centralization for the evolving collection of functional packages. Finally, there is an important collective action problem to be solved in order to allow the community to make resources available under free and open licenses, as opposed to needing to pay for access to this information.

As a potential approach to these problems, we would again point to the software engineering community as a source of potential models to be adapted, specifically the evolution of the free and open source software community. In its early stages, development will likely need to be driven by networks of researchers with specific needs for information sharing and interest in making that information freely available to others. As some of the packages of devices developed by these networks become useful, the marketplace enabled by easy publication and downloading will incentivize a wider group to begin using those packages in order to make their own work easier. This second wave of users will spread information and encourage others to use the packages, but will not generally contribute to their development, driving growth and also crises in which the growing user base stresses the resources available to the initial developers. Investment by governments or non-profit organizations may help to sustain development for some time, but ultimately , the market value proposition that drives growth should be able to be harnessed to obtain resources from industry to professionalize infrastructure and core packages in a pre-competitive space, similar to how core resources such as Linux, GitHub and Python are supported in the free and open software world.

## 6. Conclusions

Many practitioners of synthetic biology recognize the value that standardizing systems-level engineering could have on the development of biological systems and are eager to exploit the potential it has to democratize access to complex biotechnology and effect transformative change in a broad range of sectors and application areas such as healthcare, manufacturing, energy, agriculture and environmental sustainability. To date, however, the realization of that potential has proven elusive, in large part because the information required to effectively engineer with biological parts and devices has been inaccessible, insufficient or incomplete.

The Functional Synthetic Biology framework presented here lays the groundwork for a shift in orientation from the sequence-centric tools and representations that typify the field today to a focus on functionality that will transform the way synthetic biology is practiced in the future. This represents a critical step forward along the path to achieving systems-level engineering, with improvements in data sharing leading to increases in flexibility and predictability, which in turn open up opportunities for enhanced collaboration and dissemination. It is our hope that this paper will encourage others to build on the framework presented, join us on our journey to transform the practice of synthetic biology using the roadmap we have laid out as a guide and help mobilize the resources that will be required to bring that journey to a successful conclusion.

## Data Availability

Not applicable.
